# The prognostic analysis of different metastatic patterns in advanced liver cancer patients: A population based analysis

**DOI:** 10.1371/journal.pone.0200909

**Published:** 2018-08-13

**Authors:** Jie Chen

**Affiliations:** Department of Emergency, Zhejiang Provincial People’s Hospital, People’s Hospital of Hangzhou Medical College, Hangzhou, Zhejiang Province, People’s Republic of China; University of South Alabama Mitchell Cancer Institute, UNITED STATES

## Abstract

**Background:**

The prognostic impact of different distant metastases pattern in liver cancer is unexplored still now. The aim of this study is to analyze the metastasis patterns and prognosis differences for patients with stage IV liver cancers.

**Methods:**

A SEER analysis was performed. Overall survival and cancer-specific survival were calculated by the Kaplan-Meier method. Multivariable Cox regression models were used to further analyze survival outcome and other prognostic factors.

**Results:**

A total of 37526 eligible cases were retrieved in the Surveillance, Epidemiology, and End Results database. Among these patients, stage of IV liver cancer accounted for 14.80% (5555/37526) at initial diagnosis. Patients who suffered bone, brain or lung metastasis occupied 55.61% (3089/5555). Comparing with other two single metastases, the patients with brain metastasis exhibited worst overall survival whose mean of survival was 4.758 months. Multivariate analysis with Cox hazard regression model showed that metastatic site was an independent prognostic factor of overall survival and cancer-specific survival in patients with single metastasis (P<0.05). The results of univariate analysis showed that metastatic pattern was significantly correlated with overall survival (P = 0.038) and cancer-specific survival (P = 0.035) of patients with two sites.

**Conclusions:**

Lung was the most common site of single metastasis for liver cancers. Patients with bone metastasis had best survival outcome comparing with other two distant metastases. Patients with two metastatic sites, where one of them is the lung tends to have a slight trend to a worse outcome.

## Introduction

Primary liver cancer (LCa) is estimated to be one of the most common cancers worldwide, as well as one of the most common causes of cancer-related mortality. In the United States, LCa represents the fifth most common cancer deaths in men and the eighth most common deaths in women [1]. Hepatitis C virus infection is the leading cause of LCa in the United States, whereas hepatitis B virus infection is the leading cause worldwide, particularly in regions of Asia and Africa [2]. Other relevant risk factors consist of heavy alcohol drinking, tobacco, overweight, metabolic syndrome, and selected aspects of diet [3–7]. In countries without nationwide LCa surveillance programs, up to 30%-35% of patients present with macrovascular invasion and/or extrahepatic spread at initial diagnosis and the most common sites of distant metastasis are lungs, bones and adrenal glands [8–11]. The survival time of untreated metastatic LCa rarely exceed 62 days (IQR, 31–153 days) [12]. As a multi-kinase inhibitor, sorafenib, has become the standard treatment for metastatic LCa patients[13]. However, among patients with decompensated cirrhosis, the median survival benefit was 31 days, and it was not cost-effective (ICER, $224,914 per life year gained) [12]. One of the main issues related to the cost of LCa is that treatments and testing do not equate with equivalent benefit. Hence, further understanding of outcome of LCa, especially metastatic LCa, might help make suitable medical decision and save the unnecessary expend on the LCa.

However, to date little attention has been focused on the prognostic significance of distant metastatic patterns of LCa patients at the initial diagnosis. Since knowledge of prognosis of these patterns is crucial for pre-treatment evaluation, our study aimed to describe the distant metastatic site, frequency of occurrence and pattern of these metastases based on a large population using SEER database. In addition, with the considerable advances in treatment for LCa, such as surgical resection, percutaneous ablation, transcatheter arterial chemoembolization (TACE), and liver transplantation, the survival of LCa patients has improved much in recent years[14–18]. As a result, likelihood of encountering distant metastases from early stage LCa is rising. Hence, understanding the prognosis of different distant metastatic pattern in LCa patients at initial diagnosis would provide more evidence for precise medicine for metastatic LCa patients developed from early stages after diverse treatments.

## Materials and methods

### Database and patient selection

The Surveillance, Epidemiology, and End Results (SEER) program is a United States population-based cancer registry that began in 1973 and is supported by both the National Cancer Institute and Centers for Disease Control and Prevention. A total of 18 population-based cancer registries in the United States were included in the current SEER database. We totally choose 37526 cases according to the following criteria: (1) pathologically confirmed LCa with active follow-up and confirmed age. (2) the year of diagnosis from 2010 to 2014; (3) enrolled patients should have confirmed metastatic information of bone, brain, and lung. Patients with benign or borderline tumors, unknown age and unknown survival months were excluded. SEER*Stat software (SEER*Stat 8.2.3) was used to extract the data.

### Statistical analysis

Frequency distribution of demographic and clinicalpathogical characteristics across metastatic groups were compared using Pearson’ s Chi-square tests. Primary end points include overall survival (OS; defined as the time from diagnosis till death due to any reason) and cancer-specific survival (CSS; defined as the time from diagnosis till death due to LCa). The Kaplan-Meier analyses were used to generate the survival curves and the Log Rank test was applied to analyze the differences among the curves. Adjusted HRs with 95% CIs were calculated using Cox proportional hazard regression models to estimate prognostic factors. All statistical tests were 2-sided, and P<0.05 was considered statistically significant. The statistical software SPSS 22.0 was utilized for all data analyses.

## Results

### Patients’ characteristics and frequency difference of different metastases pattern

A total of 37526 eligible cases were retrieved in the SEER database. Among these patients, stage of IV LCa accounted for 14.80%(5555/37526) at initial diagnosis. The SEER database only offered metastatic information of bone, brain and lung. Patients who suffered metastasis to either one of these four organs occupied 55.61%(3089/5555).

Table 1 summarised the distribution of clinical characteristics of these patients. Age at diagnosis had substantive differences across the bone metastasis and lung metastasis groups (both, P<0.05). The distribution of race, histological grade and insurance status among patients with bone metastasis and without bone metastasis was significantly distinguishing (P<0.05). Similar phenomenon was observed in lung metastasis (P<0.05) while not in brain metastasis (P>0.05). As shown in Table 1, there were a series of significant differences among the three groups of patient samples including nodal status, T-stage, and marital status (all, P<0.05).

**Table 1:** Clinical features and metastasis sites.

Features	Bone metastasis (%)		P	Brain metastasis (%)		P	Lung metastasis (%)		P
	No	Yes		No	Yes		No	Yes	
Age									
Mean (years)	63.60	64.45	0.011	63.63	62.63	0.391	63.72	62.36	<0.001
SD	12.52	11.29		12.47	10.93		12.28	14.84	
Race									
White	21901(95.5)	1025(4.5)	<0.001	22805(99.6)	86(0.4)	0.340	21463(93.8)	1417(6.2)	<0.001
Black	4202(94.3)	254(5.7)		4425(99.6)	18(0.4)		4082(92.1)	351(7.9)	
Other	5030(96.6)	177(3.4)		5182(99.7)	13(0.3)		4826(92.8)	372(7.2)	
Nodal status									
Nx	1921(88.6)	246(11.4)	<0.001	2124(99.0)	22(1.0)	<0.001	1809(84.1)	343(15.9)	<0.001
N0	24731(97.0)	778(3.0)		25428(99.7)	64(0.3)		24390(95.8)	1072(4.2)	
N1	1846(88.4)	242(11.6)		2070(99.5)	11(0.5)		1738(83.4)	347(6.6)	
T-stage									
Tx	2462(87.8)	341(12.2)	<0.001	2753(99.1)	26(0.9)	<0.001	2397(85.9)	395(14.1)	<0.001
T1	12401(97.9)	264(2.1)		12632(99.8)	24(0.2)		12275(97.1)	369(2.9)	
T2	6157(97.7)	148(2.3)		6287(99.8)	14(0.2)		6133(97.4)	162(2.6)	
T3	6502(93.9)	426(6.1)		6894(99.6)	27(0.4)		6287(90.9)	628(9.1)	
T4	963(92.8)	75(7.2)		1032(99.4)	6(0.6)		827(80.4)	201(19.6)	
Histological grade									
Well	3250(97.5)	84(2.5)	<0.001	3319(99.7)	9(0.3)	0.148	3235(97.2)	93(2.8)	<0.001
Moderate	5095(97.2)	143(2.8)		5219(99.7)	17(0.3)		4988(95.6)	232(4.4)	
Poorly	2615(94.5)	151(5.5)		2757(99.6)	12(0.4)		2455(88.9)	306(11.1)	
Undifferentiated	269(95.7)	12(4.3)		278(98.9)	3(1.1)		249(88.0)	34(12.0)	
Marital status									
Married	15198(95.8)	663(4.2)	<0.001	15800(99.7)	42(0.3)	0.008	14843(93.8)	983(6.2)	0.002
Single	7559(91.4)	715(8.6)		15039(99.6)	67(0.4)		14032(92.9)	1069(7.1)	
Insurance									
Insured	35901(95.5)	1690(4.5)	0.002	30411(99.7)	102(0.3)	0.055	28548(93.6)	1941(6.4)	<0.001
Uninsured	1315(93.7)	88(6.3)		1392(99.4)	9(0.6)		1244(89.1)	152(10.9)	

SD: Standard deviation

The metastatic pattern of LCa was presented in Table 2. There were 7 possible metastatic forms, including 3 single metastases and 4 combinations of metastases. Among patients with single metastasis, we found that lung was still the most common site of metastasis for LCa (30.20%), followed by bone (17.8%) and brain (0.70%) metastasis. As for two sites, the combination of bone and lung metastases occupied most achieving to 5.65%.

**Table 2:** Frequencies of combination metastasis.

Features	Bladder cancer	
	Number	Percentage (%)
One site		
Only bone	987	17.8
Only brain	39	0.70
Only lung	1676	30.2
Two sites		
Lung and bone	314	5.65
Lung and brain	24	0.43
Bone and brain	24	0.43
Three sites		
Bone and brain and lung	18	0.32

### Univariate and multivariate survival analysis of patients with four single metastases

**Fig 1:**
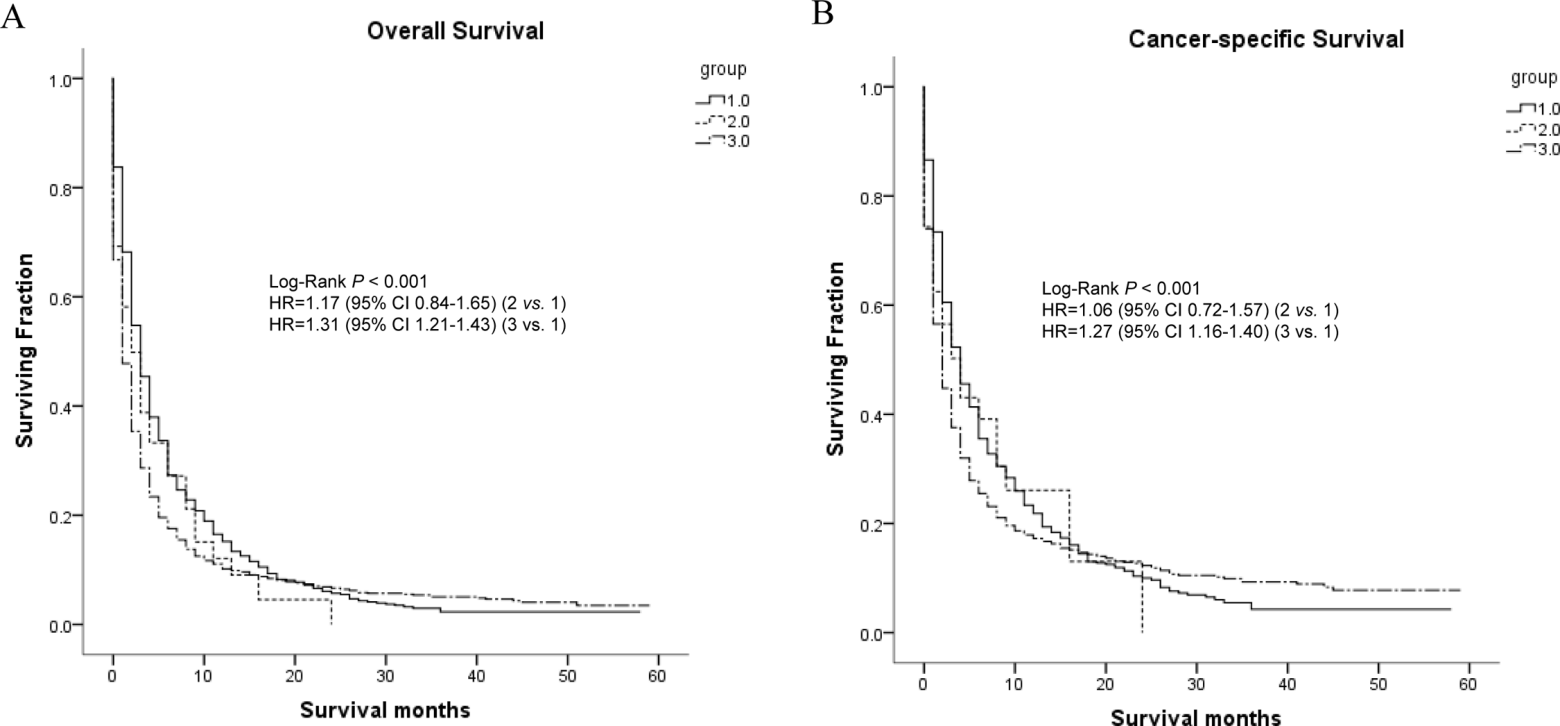
Kaplan-Meier curves and Log-rank test for overall survival (A) and cancer-specific survival (B) according to different metastasis (only one site). **Note**: 1 = Bone metastasis; 2 = Brain metastasis; 3 = Lung metastasis.

Moreover, we conducted univariate analysis (Table 3) to evaluate the impact of single metastases and baseline characteristics on OS and CCS. As was shown, metastatic sites had significant impact on OS and CCS (both, P<0.05). Comparing with other two single metastases, the patients with brain metastasis exhibited worst OS whose mean of survival was 4.758 months. As for CCS, patients with lung metastasis exhibited worst OS whose mean of survival was 7.875 months. Further multivariate analysis with Cox hazard regression model showed that metastatic site was an independent prognostic factor for both OS and CCS (P<0.05) (Table 4). Univariate analysis showed that N-classification was significantly associated with OS (P<0.05) and CCS (P<0.05) while only with OS (P<0.05) in multivariate model. In addition, other factors including T-stage, differentiated grade and marital status were all distinctly correlated with OS and CCS in both univariate and multivariate model (all, P<0.05). Fig 1 exhibited the survival curves generated by Kaplan-Meier analyses using univariate model.

**Table 3:** Univariate survival analysis of patients with three single metastases.

Risk Factors	Overall Survival			Cancer-specific Survival		
	Mean of survival months	95% CI	P	Mean of survival months	95% CI	P
Metastasis site			<0.001			<0.001
bone metastasis	6.692	(5.944, 7.441)		8.944	(7.796, 10.092)	
brain metastasis	4.758	(2.715, 6.801)		9.075	(4.218, 13.932)	
lung metastasis	5.683	(4.999, 6.367)		7.875	(6.695, 9.055)	
Race			0.808			0.444
White	6.161	(5.529, 6.793)		8.703	(7.587, 9.819)	
Black	5.424	(4.364, 6.483)		7.560	(5.853, 9.267)	
Other	6.277	(4.926, 6.544)		7.581	(5.788, 9.374)	
N-classification			0.002			0.002
N0	6.060	(5.428, 6.693)		9.088	(8.013, 10.163)	
N1	4.191	(3.391, 4.991)		5.941	(4.689, 7.193)	
T-stage			<0.001			<0.001
T1	6.966	(5.828, 8.105)		10.277	(8.505, 12.048)	
T2	7.531	(5.767, 9.294)		10.056	(7.669, 12.443)	
T3	4.752	(4.143, 5.362)		6.646	(5.716, 7.575)	
T4	4.296	(3.119, 5.473)		5.296	(3.811, 6.781)	
Differentiated grade			<0.001			<0.001
Well	9.590	(6.620, 12.531)		13.786	(9.487, 18.086)	
Moderate	8.286	(6.519, 10.053)		12.746	(9.898, 15.593)	
Poorly	3.449	(2.570, 4.328)		5.628	(3.786, 7.470)	
Undifferentiated	10.827	(4.652, 17.003)		17.239	(8.584, 25.893)	
Marital status			0.003			0.002
Married	6.488	(5.583, 7.393)		9.182	(7.833, 10.531)	
Single	5.063	(4.371, 5.755)		7.110	(6.038, 8.182)	
Insurance status			0.025			0.530
Insured	5.793	(5.379, 6.567)		8.403	(7.512, 9.293)	
Uninsured	4.389	(2.524, 6.253)		6.404	(3.486, 9.322)	

**Table 4:** Multivariate survival analysis of patients with three single metastases.

Risk Factors	Overall Survival			Cancer-specific Survival		
	HR	95% CI	P	HR	95% CI	P
Metastasis site			<0.001			<0.001
bone metastasis	1		Ref	1		Ref
brain metastasis	1.194	(0.849, 1.680)	0.309	1.068	(0.720, 1.586)	0.743
lung metastasis	1.391	(1.276, 1.517)	<0.001	1.322	(1.201, 1.456)	<0.001
Race 0.597						0.870
White	1		Ref	1		Ref
Black	1.052	(0.940, 1.177)	0.380	1.055	(0.932, 1.194)	0.400
Other	0.966	(0.858, 1.086)	0.561	1.007	(0.885, 1.146)	0.915
N-classification			0.039			0.138
N0	1		Ref	1		Ref
N1	1.135	(1.006, 1.281)	0.039	1.107	(0.968, 1.265)	0.138
T-stage			0.003			0.003
T1	1		Ref	1		Ref
T2	0.978	(0.825, 1.159)	0.794	1.010	(0.836, 1.221)	0.918
T3	1.204	(1.066, 1.359)	0.003	1.237	(1.080, 1.417)	0.002
T4	1.189	(0.994, 1.421)	0.058	1.341	(1.105, 1.627)	0.003
Differentiated grade			<0.001			<0.001
Well	1		Ref	1		Ref
Moderate	1.037	(0.826 1.302)	0.756	1.001	(0.774, 1.303)	0.975
Poorly	1.644	(1.314, 2.057)	<0.001	1.720	(1.336, 2.216)	<0.001
Undifferentiated	1.291	(0.881, 1.892)	0.191	1.233	(0.794, 1.914)	0.351
Marital status			0.008			0.011
Marrried	1		Ref	1		Ref
Single	1.122	(1.031, 1.222)	0.008	1.131	(1.028, 1.244)	0.011
Insurance status			0.016			0.031
Insured	1		Ref	1		Ref
Uninsured	1.222	(1.039, 1.438)	0.016	1.218	(1.019, 1.456)	0.031

### Univariate and multivariate survival analysis of patients with different combinations of metastases

**Fig 2:**
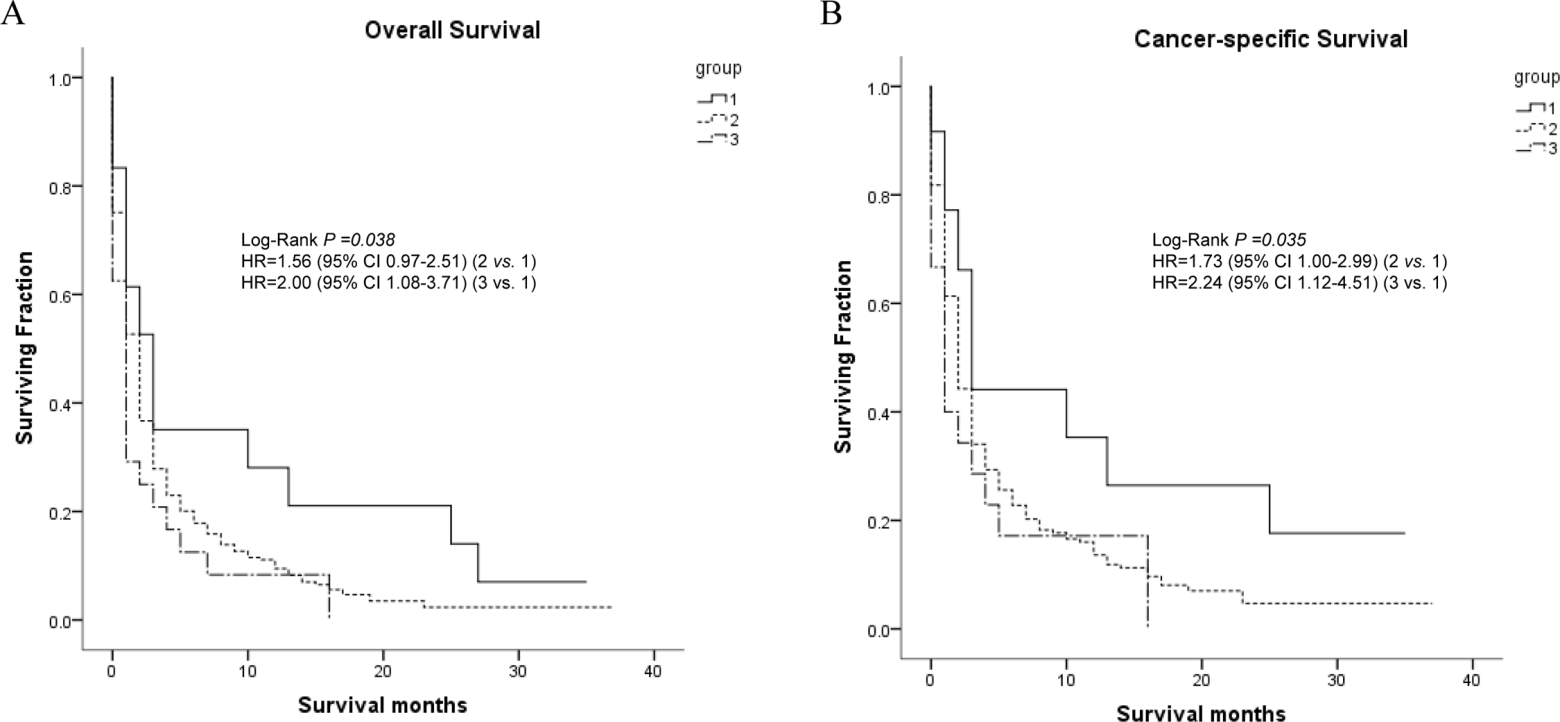
Kaplan-Meier curves and Log-rank test for overall survival (A) and cancer-specific survival (B) according to different metastasis (two sites). **Note**:1 = Bone and brain metastasis; 2 = Bone and lung metastasis; 3 = Brain and lung metastasis.

Patients with two metastatic sites had 3 forms, including bone with brain metastasis, bone with lung metastasis and brain with lung metastasis. The univariate analysis results showed that metastatic pattern was associated with OS (Bone and brain metastasis: 8.640 months; Bone and lung metastasis: 4.107 months; Brain and lung metastasis: 2.542 months; P = 0.038) and CCS (Bone and brain metastasis: 11.437 months; Bone and lung metastasis: 5.553 months; Brain and lung metastasis: 3.810 months; P = 0.035) of patients (S1 Table). In multivariate model analysis, the results showed that the patients with combination of brain and lung had the worse OS than combination of bone and brain (HR, 2.002; 95% CI, 1.081–3.707; P = 0.027). As for CCS, the patients with bone and brain had the best outcome in the three patterns with two sites (Bone and lung: HR, 1.730, 95% CI, 1.002–2.986, P = 0.049; Brain and lung: HR, 1.322, 95% CI, 1.117–4.508, P = 0.023) (S2 Table). Survival curves for these 3 forms of metastases using Kaplan-Meier method was shown in Fig 2.

## Discussion

In contrast to declining trends of other common cancers in mortality, death rates rose from 2010 to 2014 by almost 3% per year for LCa, particularly for metastatic patients with 5-year relative survival rates of 3.1%[1,19]. Metastatic LCa represents a heterogeneous disease; clinical outcomes are highly variable and depend on the underlying tumor biology and patient characteristics[20,21]. The prognostic influence of metastasis at initial diagnosis and factors associated with specific organ involvement have been understudied[22,23]. Nevertheless, there was still no study that focused on the prognosis of different distant metastases pattern in LCa. A better understanding of patterns of metastases would be valuable to assess prognosis, select appropriate treatments, and determine disease monitoring.

In our retrospective study, we observed that LCa predominantly metastasize to lung in single metastasis which is in line with previous studies[23–25]. Studies have already indicated that brain is the least common distant metastatic organ in LCa patients[26,27]. Consistent with these studies, our results also showed that brain also was the least common metastatic site in all LCa. As for bone metastasis, we found this pattern occupied 17.8% of all, which is lower than 25.5% to 38.5% reported by previous studies[28,29]. Referring to demographics and clinical features of patients, we found that that elder people seemed to suffer bone and lung metastases more frequently at diagnosis except for brain metastasis. We also found that black people was associated with more involvement of bone, and lung metastases than white people. Of note, we found that uninsured patients had more metastases to bone, brain, and lung than insured patients. Similar phenomenon was observed in aspect of marital status. In addition, patients with lymph node positive, high tumor stage and poorly differentiated histological grade were more inclined to suffer distant metastases.

We also made some findings in survival analysis. First, in multivariate survival analysis of patients with three single metastases, the OS and CCS of isolated bone metastases were the best. Outcome of patients with lung metastasis was worse than patients with bone metastasis. Hence, the presence of lung metastasis was an indicator of poor survival for primary LCa. The multivariate analysis revealed that patients with lymph node positive plus either one of other three metastases had worse overall survival than those with only distant organ metastases. Several studies reported that tumor cells metastasizing only to lymph nodes might specific epigenetic modifications that could prevent them spread to visceral organs[30,31]. Recent two studies from American and Austria have confirmed that tumor cells in lymph node could spread into vascular circulation and metastasize to distant organ[32,33]. This indicated that the tumor cells in lymph node of patients with both lymph node and distant organ metastases had a more aggressive phenotype[34]. Hence, we should focus on both distant organ metastases and lymph node status in clinical practice. In addition, poorer differentiated grade was associated with worse OS and CCS.

Some LCa patients developed more than one metastatic site, and few studies have reported on the combination of metastasis in these patients. In our study, metastasis to two sites most commonly involved the lung and bone achieving to 5.6%. In survival analysis of patients with combination of two metastatic sites, we found that patients with bone metastases plus either one of other two organ metastases had better OS and CCS than those without bone metastases. This implied that visceral metastases resulted in shorter survival than bone metastases, which was also confirmed in univariate and multivariate survival analysis of patients with only one metastasis. Our study also indicated that patients with two metastatic sites, where one of them is the lung tends to have a slight trend to a worse outcome. We therefore believe that it is important to classify patients with two metastatic sites involving the lung in order to improve the prognosis or treatment value in these specific patients.

Despite valuable findings above, there are several limitations in our study due to the retrospective nature. First, metastases to only the brain, bone and lung were included in the study. Metastasis to the adrenal glands or other metastasis sites may also influence the prognosis of LCa. Second, due to the absence of information on chemotherapy or targeted therapy included in the SEER database, their effects on survival could not be evaluated. This may cause a certain bias in our results. Third, some types of imaging that was done for patients might ignore potential distant metastases. The SEER database didn’t offer the information of the type of imaging, which could lead to bias.

## Conclusion

Lung is the most common site of single metastasis for LCa. Importantly, our results identify that metastases to the lung alone or in combination with other organs indicated a worse outcome for patients with distant metastasis. Information on the prognostic impact of different sites of metastases would provide more evidence for precise medicine and individualized therapy.

## Supporting information

S1 Table Univariate survival analysis of patients with two metastatic sites. (DOCX)

S2 Table Multivariate survival analysis of patients with two metastatic sites. (DOCX)

## References

[1] Siegel RL, Miller KD, Jemal A. Cancer Statistics, 2017. CA: a cancer journal for clinicians. 2017;67(1):7–30. 10.3322/caac.21387 28055103

[2] Bertuccio P, Turati F, Carioli G, Rodriguez T, La Vecchia C, Malvezzi M, et al Global trends and predictions in hepatocellular carcinoma mortality. J Hepatol. 2017;67(2):302–9. 10.1016/j.jhep.2017.03.011 28336466

[3] Turati F, Galeone C, Rota M, Pelucchi C, Negri E, Bagnardi V, et al Alcohol and liver cancer: a systematic review and meta-analysis of prospective studies. Annals of oncology: official journal of the European Society for Medical Oncology. 2014;25(8):1526–35. 10.1093/annonc/mdu020 24631946

[4] Chuang SC, La Vecchia C, Boffetta P. Liver cancer: descriptive epidemiology and risk factors other than HBV and HCV infection. Cancer letters. 2009;286(1):9–14. 10.1016/j.canlet.2008.10.040 19091458

[5] Turati F, Trichopoulos D, Polesel J, Bravi F, Rossi M, Talamini R, et al Mediterranean diet and hepatocellular carcinoma. J Hepatol. 2014;60(3):606–11. 10.1016/j.jhep.2013.10.034 24240052

[6] Shivappa N, Hebert JR, Polesel J, Zucchetto A, Crispo A, Montella M, et al Inflammatory potential of diet and risk for hepatocellular cancer in a case-control study from Italy. The British journal of nutrition. 2016;115(2):324–31. 10.1017/S0007114515004419 26556602

[7] Polesel J, Talamini R, Montella M, Maso LD, Crovatto M, Parpinel M, et al Nutrients intake and the risk of hepatocellular carcinoma in Italy. European journal of cancer (Oxford, England: 1990). 2007;43(16):2381–7. 10.1016/j.ejca.2007.07.012 17719221

[8] Katyal S, Oliver JH, Peterson MS, Ferris JV, Carr BS, Baron RL. Extrahepatic metastases of hepatocellular carcinoma. Radiology. 2000;216(3):698–703. 10.1148/radiology.216.3.r00se24698 10966697

[9] Uka K, Aikata H, Takaki S, Shirakawa H, Jeong SC, Yamashina K, et al Clinical features and prognosis of patients with extrahepatic metastases from hepatocellular carcinoma. World J Gastroenterol. 2007;13(3):414–20. 10.3748/wjg.v13.i3.414 17230611 PMC4065897

[10] Villanueva A, Hernandez-Gea V, Llovet JM. Medical therapies for hepatocellular carcinoma: a critical view of the evidence. Nature reviews. Gastroenterology & hepatology. 2013;10(1):34–42. 10.1038/nrgastro.2012.199 23147664

[11] Mittal S, Kanwal F, Ying J, Chung R, Sada YH, Temple S, et al Effectiveness of surveillance for hepatocellular carcinoma in clinical practice: A United States cohort. J Hepatol. 2016;65(6):1148–54. 10.1016/j.jhep.2016.07.025 27476765 PMC5322857

[12] Parikh ND, Marshall VD, Singal AG, Nathan H, Lok AS, Balkrishnan R et al Survival and cost-effectiveness of sorafenib therapy in advanced hepatocellular carcinoma: An analysis of the SEER-Medicare database. Hepatology. 2017;65(1):122–33. 10.1002/hep.28881 27770556

[13] Llovet JM, Ricci S, Mazzaferro V, et al Sorafenib in advanced hepatocellular carcinoma. The New England journal of medicine. 2008;359(4):378–90. 10.1056/NEJMoa0708857 18650514

[14] Shiina S, Teratani T, Obi S, Hilgard P, Gane E, Blanc JF, et al A randomized controlled trial of radiofrequency ablation with ethanol injection for small hepatocellular carcinoma. Gastroenterology. 2005;129(1):122–30. 16012942 10.1053/j.gastro.2005.04.009

[15] Mazzaferro V, Regalia E, Doci R, Andreola S, Pulvirenti A, Bozzetti F, et al Liver transplantation for the treatment of small hepatocellular carcinomas in patients with cirrhosis. The New England journal of medicine. 1996;334(11):693–9. 10.1056/NEJM199603143341104 8594428

[16] Arii S, Yamaoka Y, Futagawa S, Inoue K, Kobayashi K, Kojiro M, et al Results of surgical and nonsurgical treatment for small-sized hepatocellular carcinomas: a retrospective and nationwide survey in Japan. The Liver Cancer Study Group of Japan. Hepatology. 2000;32(6):1224–9. 10.1053/jhep.2000.20456 11093728

[17] Shiina S, Tagawa K, Niwa Y, Unuma T, Komatsu Y, Yoshiura K, et al Percutaneous ethanol injection therapy for hepatocellular carcinoma: results in 146 patients. AJR. American journal of roentgenology. 1993;160(5):1023–8. 10.2214/ajr.160.5.7682378 7682378

[18] Llovet JM, Real MI, Montana X, Planas R, Coll S, Aponte J, et al Arterial embolisation or chemoembolisation versus symptomatic treatment in patients with unresectable hepatocellular carcinoma: a randomised controlled trial. Lancet (London, England). 2002;359(9319):1734–9. 10.1016/s0140-6736(02)08649-x 12049862

[19] Ryerson AB, Eheman CR, Altekruse SF, Ward JW, Jemal A, Sherman RL, et al Annual Report to the Nation on the Status of Cancer, 1975–2012, featuring the increasing incidence of liver cancer. Cancer. 2016;122(9):1312–37. 10.1002/cncr.29936 26959385 PMC4840031

[20] Bosch FX, Ribes J, Borras J. Epidemiology of primary liver cancer. Seminars in liver disease. 1999;19(3):271–85. 10.1055/s-2007-1007117 10518307

[21] Tandon P, Garcia-Tsao G. Prognostic indicators in hepatocellular carcinoma: a systematic review of 72 studies. Liver Int. 2009;29(4):502–10. 10.1111/j.1478-3231.2008.01957.x 19141028 PMC2711257

[22] Wu W, He X, Andayani D, Yang L, Ye J, Li Y, et al Pattern of distant extrahepatic metastases in primary liver cancer: a SEER based study. J Cancer. 2017;8(12):2312–8. 10.7150/jca.19056 28819435 PMC5560150

[23] Lee JI, Kim JK, Kim DY, Ahn SH, Park JY, Kim SU, et al Prognosis of hepatocellular carcinoma patients with extrahepatic metastasis and the controllability of intrahepatic lesions. Clin Exp Metastasis. 2014;31(4):475–82. 10.1007/s10585-014-9641-x 24496959

[24] Sawabe M, Nakamura T, Kanno J, Kasuga T. Analysis of morphological factors of hepatocellular carcinoma in 98 autopsy cases with respect to pulmonary metastasis. Acta pathologica japonica. 1987;37(9):1389–404. 2825465 10.1111/j.1440-1827.1987.tb02261.x

[25] Natsuizaka M, Omura T, Akaike T, Kuwata Y, Yamazaki K, Sato T, et al Clinical features of hepatocellular carcinoma with extrahepatic metastases. J Gastroenterol Hepatol. 2005;20(11):1781–7. 10.1111/j.1440-1746.2005.03919.x 16246200

[26] Seinfeld J, Wagner AS, Kleinschmidt-DeMasters BK. Brain metastases from hepatocellular carcinoma in US patients. J Neurooncol. 2006;76(1):93–8. 10.1007/s11060-005-4175-3 16402279

[27] Del Ben M, Caporale A, Feole K, Alessandri C, Angelico F. Intracranial hemorrage due to brain metastases in an Italian HCV patient with hepatocellular carcinoma. Journal of experimental & clinical cancer research: CR. 2003;22(4):641–4. 15053309

[28] Fukutomi M, Yokota M, Chuman H, Harada H, Zaitsu Y, Funakoshi A, et al Increased incidence of bone metastases in hepatocellular carcinoma. European journal of gastroenterology & hepatology. 2001;13(9):1083–8. 11564960 10.1097/00042737-200109000-00015

[29] Uchino K, Tateishi R, Shiina S, Kanda M, Masuzaki R, Kondo Y, et al Hepatocellular carcinoma with extrahepatic metastasis: clinical features and prognostic factors. Cancer. 2011;117(19):4475–83. 10.1002/cncr.25960 21437884

[30] Barekati Z, Radpour R, Lu Q, Bitzer J, Zheng H, Toniolo P, et al Methylation signature of lymph node metastases in breast cancer patients. BMC Cancer. 2012;12:244 10.1186/1471-2407-12-244 22695536 PMC3437205

[31] Briganti A, Passoni NM, Abdollah F, Nini A, Montorsi F, Karnes RJ. Treatment of lymph node-positive prostate cancer: teaching old dogmas new tricks. European urology. 2014;65(1):26–7; discussion 8. 10.1016/j.eururo.2013.07.003 23886795

[32] Pereira ER, Kedrin D, Seano G, Gautier O, Meijer EFJ, Jones D, et al Lymph node metastases can invade local blood vessels, exit the node, and colonize distant organs in mice. Science (New York, N.Y.). 2018;359(6382):1403–7. 10.1126/science.aal3622 29567713 PMC6002772

[33] Brown M, Assen FP, Leithner A, Abe J, Schachner H, Asfour G, et al Lymph node blood vessels provide exit routes for metastatic tumor cell dissemination in mice. Science (New York, N.Y.). 2018;359(6382):1408–11. 10.1126/science.aal3662 29567714

[34] Furuya Y, Akakura K, Akimoto S, Ito H. Prognosis of patients with prostate carcinoma presenting as nonregional lymph node metastases. Urologia internationalis. 1998;61(1):17–21. 10.1159/000030277 9792977

