# Supplementary material for: The prognostic analysis of different metastatic patterns in advanced liver cancer patients: A population based analysis
Source: PLoS One. 2018 Aug 13;13(8):e0200909. doi: 10.1371/journal.pone.0200909 (PMC6089416; doi:10.1371/journal.pone.0200909)
Supplement: S1 Table — (DOCX) [file pone.0200909.s001.docx]

**Table S1** Univariate survival analysis of patients with two metastatic sites

| Risk Factors | Overall Survival | | | | | | | Cancer-specific Survival | | | |
| --- | --- | --- | --- | --- | --- | --- | --- | --- | --- | --- | --- |
|  | Mean of survival months | | | 95% CI | | P | | Mean of survival months | | 95% CI | P |
| Metastasis site 0.038 0.035 | | | | | | | | | | | |
| Bone and brain metastasis | | 8.640 | （3.704，13.577） | |  | | 11.437 | | （5.082，17.792） | | |
| Bone and lung metastasis | | 4.107 | （3.264，4.950） | |  | | 5.553 | | （4.299，6.808） | | |
| Brain and lung metastasis | | 2.542 | （0.735，4.348） | |  | | 3.810 | | （1.237，6.382） | | |
