# Supplementary material for: The prognostic analysis of different metastatic patterns in advanced liver cancer patients: A population based analysis
Source: PLoS One. 2018 Aug 13;13(8):e0200909. doi: 10.1371/journal.pone.0200909 (PMC6089416; doi:10.1371/journal.pone.0200909)
Supplement: S2 Table — (DOCX) [file pone.0200909.s002.docx]

**Table S2** Multivariate survival analysis of patients with two metastatic sites

| Risk Factors | Overall Survival | | | | | | | Cancer-specific Survival | | | | |
| --- | --- | --- | --- | --- | --- | --- | --- | --- | --- | --- | --- | --- |
|  | HR | | | 95% CI | | P | | HR | | 95% CI | | P |
| Metastasis site 0.082 0.069 | | | | | | | | | | | | |
| Bone and brain metastasis | | 1 |  | | Ref | | 1 | | | | Ref | |
| Bone and lung metastasis | | 1.561 | (0.973, 2.506) | | 0.065 | | 1.730 | | (1.002, 2.986) | | 0.049 | |
| Brain and lung metastasis | | 2.002 | (1.081, 3.707) | | 0.027 | | 1.322 | | (1.117, 4.508) | | 0.023 | |
